# Outcomes after Descemet membrane endothelial keratoplasty over a period of 7 years at a tertiary referral center: endothelial cell density, central corneal thickness, and visual acuity

**DOI:** 10.1007/s00417-021-05152-w

**Published:** 2021-03-15

**Authors:** Tarek Bayyoud, Faik Gelisken, Jens Martin Rohrbach, Gunnar Blumenstock, Karl Ulrich Bartz-Schmidt, Sebastian Thaler

**Affiliations:** 1 Department of Ophthalmology, University Hospital of Tübingen, Tübingen, Baden-Württemberg Germany; 2 Institute for Clinical Epidemiology and Applied Biometry, University of Tübingen, Tübingen, Baden-Württemberg Germany

**Keywords:** Descemet membrane endothelial keratoplasty, Visual acuity, Endothelial cell density, Central corneal thickness

## Abstract

**Purpose:**

To better assess clinical trajectories of patients with or without ocular comorbidity after Descemet membrane endothelial keratoplasty. Background: To report on the outcomes of eyes with differing starting conditions following surgery. Design: Retrospective study at a University Eye Hospital. Participants: 361 eyes separated into group 1 (*n*=229; eyes with endothelial disease only) and group 2 (*n*=132; eyes with additional ocular comorbid conditions, such as herpetic eye disease 18/132 (13.6%), glaucoma 16/132 (12.1%), dry age-related macular degeneration 14/132 (10.6%), epiretinal membranes 10/132 (7.6%), and wet age-related macular degeneration 9/132 (6.8%)).

**Methods:**

Consecutive eyes that underwent Descemet membrane endothelial keratoplasty over a follow-up period of up to 7 years at a tertiary referral center were reviewed. Main outcome measures were best-corrected visual acuity, postoperative complications, graft survival, central corneal thickness, and endothelial cell density.

**Results:**

Postoperative best-corrected visual acuity at year 1 improved in both groups significantly (Wilcoxon signed rank test: group 1, *p* =.002; .63 to .23 logMAR; group 2, *p* <.001; 1.15 to .87 logMAR) with a group difference in favor of group 1 (*p* =.009, Mann-Whitney-Wilcoxon). A decrease of the endothelial cell density and central corneal thickness was noted at postoperative year 1 for both groups (paired *t*-tests (group 1, *p* <.001; group 2, *p* =.045) and paired *t*-tests (group 1, *p* <.001; group 2, *p* =.003). Complications were less common, and graft longevity was superior in group 1.

**Conclusion:**

Eyes with different starting conditions might experience a visual improvement and benefit from surgery. Descemet membrane endothelial keratoplasty is a valid treatment for endothelial disorders in manifold of eyes. Further long-term studies are required.

## Introduction

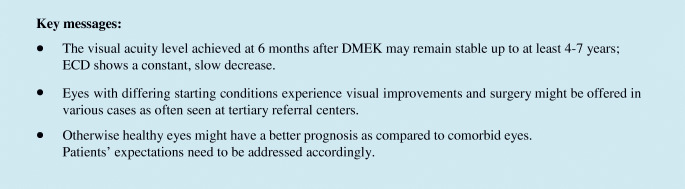



Endothelial keratoplasty has evolved over the past decade to become the standard treatment of corneal endothelial disorders [1–3]. Among the different types of lamellar keratoplastic procedures, Descemet membrane endothelial keratoplasty (DMEK) has gained increasing popularity worldwide [4]. In comparison to penetrating keratoplasty, the selective transplantation of the diseased Descemet membrane endothelial complex has proven to be superior with respect to faster visual outcome, shorter time to full rehabilitation, and lower rate of complications [5]. Currently, data concerning the outcomes for differing starting conditions is limited, although results are promising [6, 7]. Surgery seems to be more challenging under certain preoperative circumstances, as seen in multi-morbid eyes [8–11]. This study aimed to retrospectively analyze the outcomes of DMEK in eyes with or without comorbidity.

## Patients and methods

All consecutive patients who underwent DMEK at the University Eye Hospital of Tübingen between April 2010 and June 2017 were analyzed. Inclusion criteria were Fuchs endothelial dystrophy (FECD), bullous keratopathy (BK), and failed corneal transplant (after penetrating keratoplasty). A total of 361 eyes (294 patients) were analyzed in the study.

Group 1 was defined as eyes with no known comorbidities and no major intraocular surgeries or traumata prior to DMEK. Group 2 was defined as eyes with known comorbidities and/or major intraocular surgeries prior to DMEK. Cataract surgery qualified for both groups.

Group 1 consisted of 229 eyes of 173 patients with a mean age of 70.4 (±8.47) years (range: 48.2–91 years). The follow-up time was 37.2 months (SD 20; range 1–86). One hundred fifty-nine patients (92%) were diagnosed with FECD and 14 (8%) with BK. Group 2 included 132 eyes of 121 patients, with a mean age of 74.65 (±11.98) years (range: 36.4–98.4 years). The follow-up time was 23.4 months (SD 18; range 1–84). Seventy-six patients (62.8%) had a diagnosis of FECD, 42 (34.7%) BK, and 3 (2.5%) failed penetrating keratoplasty.

The research has followed the Tenets of the Declaration of Helsinki. Signed informed consent was obtained from all patients prior to surgery. The study was conducted according to requirements of the Cornea Bank of the University Hospital of Tübingen and with independent institutional review board (IRB) approval (approved by the Ethics Committee of the University of Tuebingen; Project Number: 559/2018BO2; data was analyzed anonymously).

### Graft preparation

Harvesting of the DMEK grafts was performed according to the forceps technique as described previously. ^12, 13^ After obtaining the donor globes, corneo-scleral rings were dissected and stored in organ culture media (Culture Medium I; Biochrom GmbH, Berlin, Germany). Only donor corneas with regular endothelial cell morphology and generally cell counts of 2300 cell/mm^2^ or more were used.

### Surgery

All surgeries were performed by three experienced ophthalmic surgeons. A circular 7.5-mm-diameter descemetorhexis was performed under air by scoring and stripping off the Descemet membrane endothelial complex with an incisional hook (Price Hook, Moria S.A. plc, France; Descemet incision hook, Geuder AG, Germany; Reversed Sinskey Hook, Katena Inc., USA). The graft was stained with trypan blue solution (Vision Blue; DORC, Zuidland, the Netherlands) and injected through a 2.75-mm limbal tunnel incision into the recipient anterior chamber via a custom-made injector. Iridotomy was performed for each case. After orientation of the graft—facing the iris with its endothelial side—unfolding was done using an air bubble between the recipient stroma and donor Descemet membrane (DM). Subsequently, a second air bubble, posterior to the endothelial layer, was used to attach the donor DM to the recipient’s stroma. The anterior chamber was then completely filled with air for 60 min and afterwards left with a 30–50% tamponading air bubble. Postoperatively, the patients were advised to keep a supine position for 24 h. Postoperative medications included topical steroids (dexamethasone 0.1%) four times daily for 4 weeks and antibiotics for 2 weeks (moxifloxacin). The steroid regimen was subsequently reduced to once daily after 3 months and given every other day for 1 year. After that it was advised to continue with 3–4 drops per week.

### Data collection and analysis

Primary outcome measures included best-corrected visual acuity (BCVA), endothelial cell density (ECD), and central corneal thickness (CCT); BCVA was measured by Snellen visual acuity charts and ECD/CCT by specular microscopy (noncontact autofocus specular microscope; EM-3000, TOMEY CORP., Nagoya, Japan; automated count with quality checks).

The following complications were recorded: primary graft failure (PGF), secondary graft failure (SGF), minor graft detachment, major graft detachment, and allograft rejection (AR).

PGF was defined as a cornea exhibiting clinical signs of endothelial decompensation (e.g., stromal edema, subepithelial edema, and/or bullae) after DMEK without a period of corneal clearance. SGF was defined as a graft exhibiting clinical signs of endothelial decompensation as previously mentioned and a previous period of corneal clearance (postoperative interval of at least 1 month of a clear cornea and an attached graft).

Minor graft detachment was defined as a separation of the graft of no more than one-third of the posterior stromal surface area. Major graft detachment was defined as an unattached graft of more than one-third of the posterior stromal surface area. In general, re-surgery was considered for large graft detachments. AR was defined as signs of an immune reaction (e.g., endothelial precipitates).

For statistical analysis, BCVA values were converted to a logarithm of the minimum angle of resolution (logMAR) units. To compare continuous data (BCVA change, ECD change, CCT change) between group 1 and group 2, the Mann-Whitney-Wilcoxon *U* test and the two-sample *t*-test were used, respectively. To compare graft survival between the two independent groups, the log-rank test was performed. For graphing, Kaplan-Meier curves were employed. Paired before-and-after-comparisons were tested using the Wilcoxon signed rank test and the paired *t*-test, respectively. The level of statistical significance was set to an adjusted level of .0125 for the four primary between-group comparisons and to .05 for further purely exploratory analyses. Analyses were performed using professional software (Microsoft Excel, version 2010, Microsoft and IBM SPSS, version 25, IBM).

### Criteria of comorbidity

Group 1 was defined as eyes with no known comorbidities and no major intraocular surgeries prior to DMEK but cataract surgery and endothelial disease. Group 2 was defined as eyes with known comorbidities and/or major intraocular surgeries prior to DMEK and endothelial disease (e.g., confirmed status of post-herpetic disease, status post-ocular trauma, status post-ocular surgery (e.g., pars plana vitrectomy with endotamponade, penetrating keratoplasty, glaucoma tube surgery)).

## Results

### Demographics/clinical characteristics

The data of each group is outlined in the demographics and clinical characteristics in Table 1.

**Table 1:** Demographics and clinical characteristics of Descemet membrane endothelial keratoplasty eyes

Group	Group 1	Group 2
Number of eyes	229	132
Number of patients	173	121
Mean age (±SD^a^) [range]	70.4 (±8.5) [48.2–91] years	74.7 (±12.0) [36.4–98.4] years
Female/male, *N*^b^ [%]	105/68 [60.7%/39.3%]	67/54 [55.4%/44.6%]
Laterality, R/L [%]	113/116 [49.3%/50.7%]	66/66 [50%/50%]
Phakic/pseudophakic/aphakic, *N* [%]	60/169/^c^ [26.2%/73.8% /†]	25/106/1 [18.9%/80.3%/1%]
Indication, *N* [%]		
Fuchs endothelial corneal dystrophy	213 [93%]	84 [63.6%]
Bullous keratopathy	16 [7%]	45 [34.1%]
Failed penetrating keratoplasty	c	3 [2.3%]
Average follow-up period (±SD) [range]	3.1 (±1.7) [1–7] years	2.0 (±1.5) [1–6] years

^a^Standard deviation

^b^Number

^c^By definition excluded

### Comorbidities and previous surgeries

Ocular comorbidities included in decreasing order the herpetic disease (13.6%), glaucoma (12.1%), dry age-related macular degeneration (dry AMD) (10.6%), epiretinal membranes (7.6%), wet age-related macular degeneration (wet AMD) (6.8%), retinal detachment (5.3%), and amblyopia (4.5%). Other comorbidities included trauma, uveitis, macular foramen, hereditary retinal disease, artery occlusion, vein occlusion, optic neuritis, ischemic optic neuropathy, diabetic retinopathy, fundus myopicus, corneal dystrophy, macular dystrophy, keratoconus, and vitreomacular traction. The retinal detachment and severe ocular trauma comorbidities were relatively common (*n* = 11; 8.3%). Table 2 subgroups the more common comorbidities.

**Table 2:** Comorbidities and complications

Group	*N*	Median ECL (IQR)	*p* value^a^	Graft detachment	*p* value^b^
Group 1	229	823 (522–1146)	0.025	16 (7.0%)	0.007
Group 2	132	632 (394–1028)		21 (15.9%)	
Herpetic disease	18	1158 (429–1481)		4/18	
Glaucoma	16	1115 (581–1121)		3/16	
Dry AMD	14	827 (232–2014)		2/14	
ERM	10	630 (630–630)		1/10	
Wet AMD	9	553 (404–827)		2/9	
RD	7	735 (707–1138)		0/7	
Amblyopia	6	486 (449–522)		3/6	
Overall	361	800 (485–1119)		37 (10.2%)	

*ECL* endothelial cell loss, *IQR* interquartile range, *AMD* age-related macular degeneration, *ERM* epiretinal membrane, *RD* retinal detachment

^a^*U* test (Mann-Whitney-Wilcoxon)

^b^Chi-squared test

^c^Specific comorbidities with more than 5 cases are shown separately

Previous surgeries included penetrating keratoplasty (*N*=3), pars plana vitrectomy (*N*=8), and glaucoma tube surgery (*N* = 7).

### Best-corrected visual acuity

**Fig. 1:**
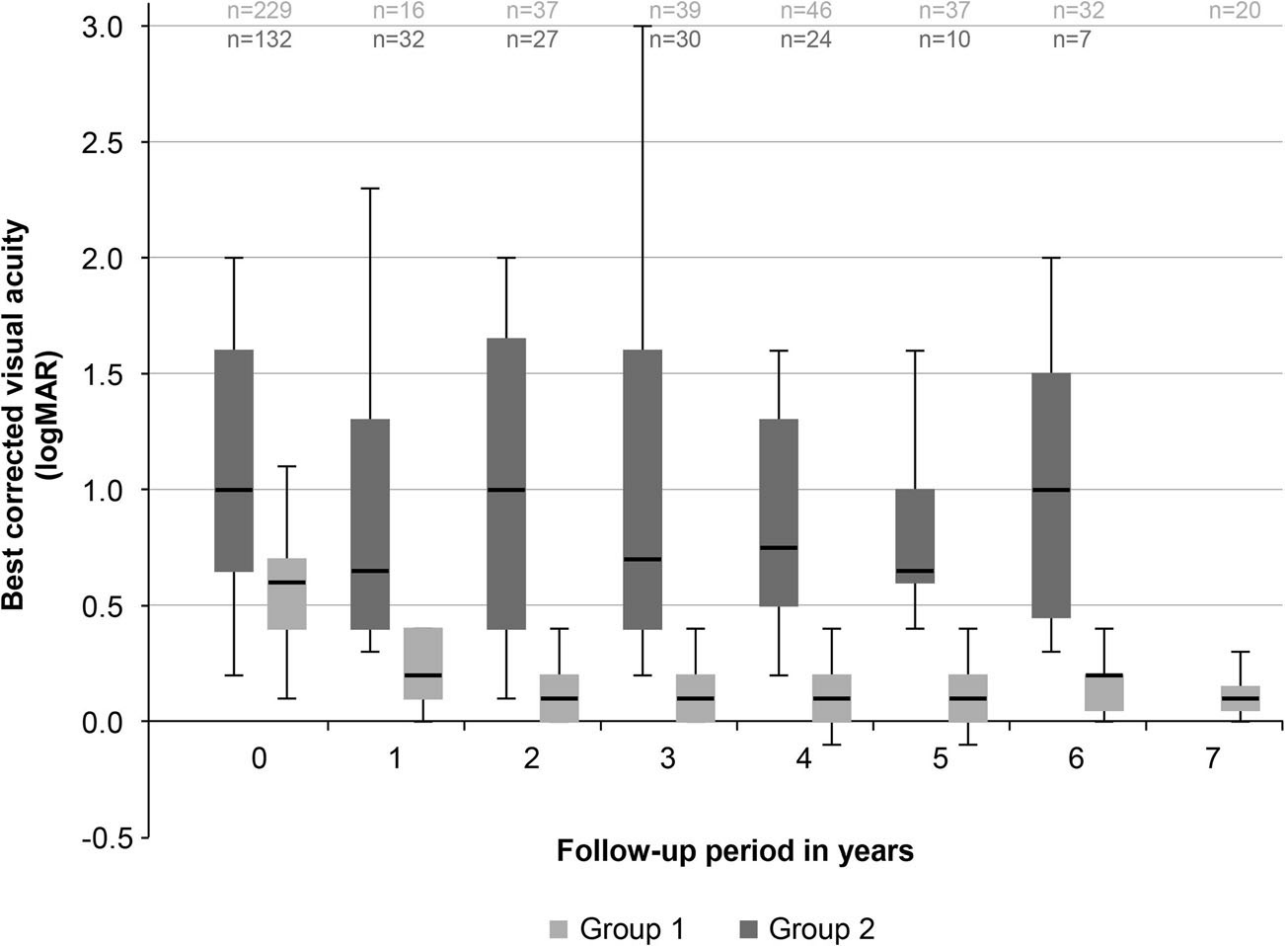
Changes of visual acuities in both groups. Best-corrected visual acuities (in logMAR) in eyes of groups 1 and 2 preoperatively and at yearly postoperative intervals (depicted as box plot graphs; *x*-axis: 0 = preoperatively; 1–7 = respective year of follow-up)

The follow-up of BCVA of each group is shown in Fig. 1.

The statistical results were significant when comparing preoperative BCVA values with postoperative ones in both groups after 1 year (Wilcoxon signed rank test: group 1, *p* = .002; group 2, *p* < .001). Group differences in the first year were statistically significant in favor of group 1 (*p* = .009, Mann-Whitney-Wilcoxon). The results were also significant in group 1 (Wilcoxon signed rank test: *p* < .001) when comparing preoperative data to data of year 7 (final year values) (*N* preoperatively and for each follow-up: 229, 16, 37, 39, 46, 37, 32, and 20). With respect to group 2, the preoperative to final year values (year 6) were not significant (Wilcoxon signed rank test: *p* = .219) (*N* preoperatively and for each follow-up: 132, 32, 27, 30, 24, 10, and 7).

In the last year of follow-up, 15 of 20 eyes of group 1 had a logMAR BCVA of 0.1 (0.8 decimal) or better.

### Endothelial cell density

In group 1, the mean ECD of the graft was 2457 (±209) cells/mm^2^ prior to surgery and decreased to 1858 (±402) cells/mm^2^ (24.4% loss) at postoperative year 1 and to 1672 (±407) cells/mm^2^ (10.0% loss; compared to the preceding, mean ECD) at year 2. At postoperative year 7, ECD was 1408 (±440) cells/mm^2^ (6.1% loss per year; mean follow-up: 37.2 months).

In group 2, the mean ECD was 2415 (±244) cells/mm^2^ prior to surgery and decreased to 1981 (±274) cells/mm^2^ (17.8% loss) at postoperative year 1 and to 1623 (±569) cells/mm^2^ (18.0% loss; compared to the preceding, mean ECD) at year 2. At postoperative year 6, ECD was 1734 (±185) cells/mm^2^ (28.2% loss; compared to the preoperative ECD with a mean follow-up period of 23.4 months).

**Fig. 2:**
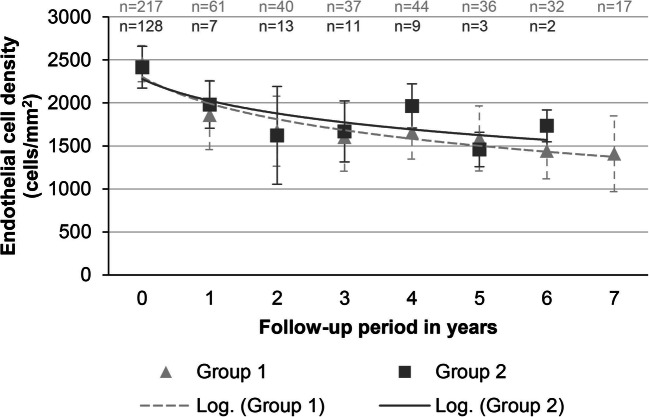
Change of the mean endothelial cell densities. Mean endothelial cell density (in cells/mm^2^) prior to surgery and at yearly postoperative intervals for each group (depicted as a logarithmic graph interpolation; *x*-axis: 0 = preoperatively; 1–7 = respective year of follow-up)

A significant decrease was noted in the postoperative years 1 and 2 (paired *t*-test: *p* < .001 and .048, respectively; *N* preoperatively and for each follow-up: 217, 61, 40, 37, 44, 36, 32, and 17) in group 1 and in the first year in group 2 (paired *t*-test: *p* = .045; *N* preoperatively and for each follow-up: 128, 7, 13, 11, 9, 3, and 2). Group differences of the first year were not significant (*p* = .230, two-sample *t*-test). The data is shown in Fig. 2.

### Central corneal thickness

In group 1, the CCT was 611 (±72) μm prior to surgery and changed to 538 (±86) μm (11.9%) after the first year of surgery and to 525 (±63) μm (2.6%; compared to the preceding, mean CCT) of the second year. At year 7, the CCT was 552 (±47) μm (9.6%; compared to the preoperative CCT; mean follow-up: 37.2 months).

In group 2, the CCT was 592 (±94) μm prior to surgery and changed to 507 (±43) μm (14.4%) after the first year of surgery and to 495 (±62) μm (2.4%; compared to the preceding, mean CCT) of the second year. At postoperative year 6, CCT was 532 (±28) μm (10.2%; compared to the preoperative CCT; mean follow-up: 23.4 months).

**Fig. 3:**
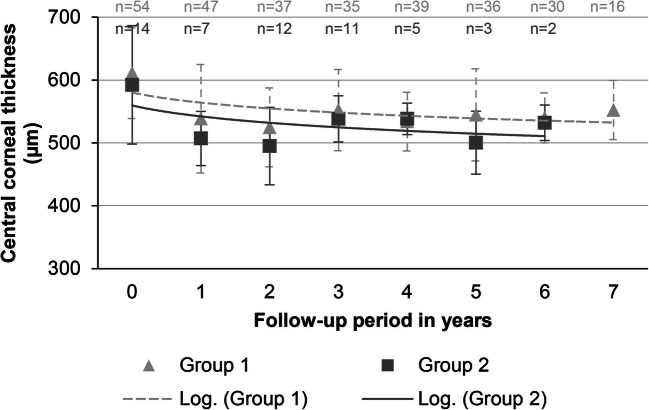
Change of the mean central corneal thickness. Mean central corneal thickness (in μm) prior to surgery and at yearly postoperative intervals for each group (depicted as a logarithmic graph interpolation; *x*-axis: 0 = preoperatively; 1–7 = respective year of follow-up)

A significant decrease was noted in the first year after surgery in both groups (paired *t*-test: groups 1 and 2: *p* < .001 and .003, respectively; *N* preoperatively and for each follow-up in group 1: 54, 47, 37, 35, 39, 36, 30, and 16; in group 2: 14, 7, 12, 11, 5, 3, and 2). Group differences of the first year were not significant (*p* = .118, two-sample *t*-test). Further data is shown in Fig. 3.

### Complications

Major graft detachments and secondary graft failures were particularly common in the comorbid group (group 2) (major graft detachments: group 2: 15.9% vs group 1: 7.0%; secondary graft failures: group 2: 15.9% vs group 1: 3.5%).

The most common complication in groups 1 and 2 included minor graft detachment (group 1: *n* = 62 [27.1%]; group 2: *n* = 29 [22%]), major graft detachment (group 1: *n* = 16 [7.0%]; group 2: *n* = 21 [15.9%]), secondary graft failure (group 1: *n* = 8 [3.5%]; group 2: *n* = 21 [15.9%)]), primary graft failure (group 1: *n* = 15 [6.6%]; group 2: *n* = 7 [5.3%]), and allograft rejection (group 1: *n* = 2 [<1%]; group 2: *n* = 2 [1.5%]).

### Additional analysis: graft survival

**Fig. 4:**
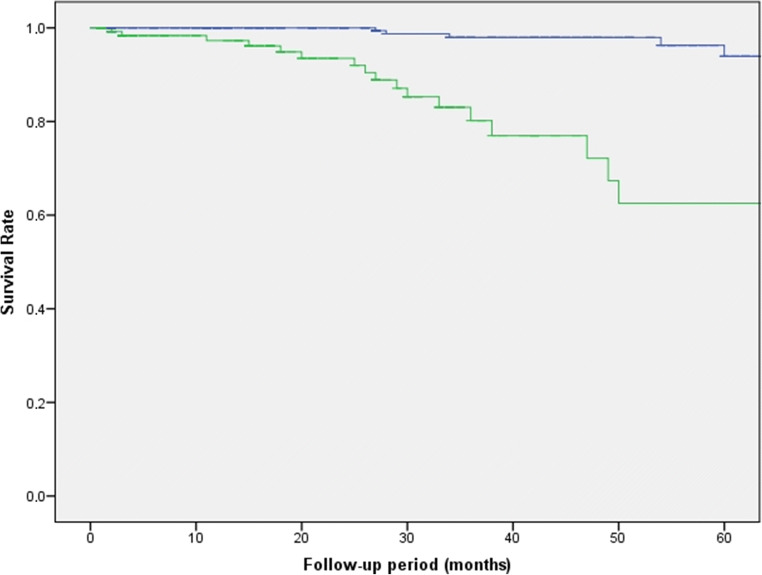
Graft survival estimation using Kaplan-Meier (blue, group 1; green, group 2)

Graft survival estimation was performed using Kaplan-Meier as shown in Fig. 4. The 5-year graft survival rate was 94% in group 1 and 62.5% in group 2 (60 months after DMEK; log-rank test: *p* < .001).

## Discussion

The aim of this study is to report on the outcomes of eyes with or without comorbidity after DMEK in a retrospective approach. This study showed differences in the clinical course of eyes with or without ocular comorbidity after DMEK. The published data following the introduction of DMEK by Melles in 2006 showed very promising short- and mid-term results [6, 12–14]. The current work analyzed, retrospectively, the outcomes in two patient cohorts to better understand the potential prognosis. To better evaluate the consecutive and extensive clinical data, eyes with comorbidity were classified as group 1 and those without as group 2. This approach was chosen to prevent potential bias and confounding parameters. Potential outcome limiting factors or individual comorbidities were reported by other studies [15–17]. Glaucoma drainage device and previously failed keratoplasty were reported to have a potential effect on graft survival rates.

The first group included eyes suffering from endothelial disease. The second group consisted of eyes having, in addition to endothelial disease, significant other ocular pathologies. To the best of our knowledge, few studies addressed the outcomes of this procedure with respect to preoperative condition and duration of follow-up [6, 18]. Results of this study may aid in preoperative decision-making and also in counseling of the affected patients.

One of the most important results was the superiority of graft survival in group 1. More than 70% of non-comorbid eyes after 7 years of follow-up showed a BCVA of 0.8 or more. The ECD counts showed a phase of stable to minimal further decline in each group. The classical rapid decrease of the ECD during the first postoperative months was masked due to the selected 12-month follow-up interval. Similarly, the CCT counts experienced an initial decline in both groups during the first postoperative year, followed by a phase of stabilization until the final year of follow-up.

The overall rate of major complications was low, except for major graft detachments and secondary graft failure in group 2. The cumulative graft survival rate was 94% in group 1 and 62.5% in group 2 at 60 months. The higher rate of graft failures in the comorbid group might be explained by a higher effort to position the graft, by coexisting ocular disease and prior ocular interventions (e.g., penetrating keratoplasty, glaucoma tubes).

Postoperative ECD and CCT developments were in accordance with published data [6, 18]. Ham et al. reported stable clinical outcomes of up to 7 years. The endothelial cells were shown to undergo a constant slow decrease, whereas the rate of complications after the first six postoperative months remained quite low (<5%). Schlögl et al. confirmed the mid-to-long-term sustainability by showing stable clinical outcomes over 5 years, including visual acuity and endothelial cell counts. The potential for full visual rehabilitation of eyes after DMEK is noted as already shown by other groups [19–21].

Graft survival and visual improvements did meet the expectations. This also holds true for eyes of group 2. These patients did exhibit a significant visual improvement albeit coexisting ocular pathologies. Graft survival estimates in non-comorbid eyes are consistent with previously published work [6, 18]. As shown for penetrating keratoplasty, comorbidities, such as trauma and the original diagnosis such as bullous keratopathy, have a negative impact on graft survival [22–24]. Trauma was one of the comorbidities in group 2, and bullous keratopathy was an indication for surgery in every third eye in group 2. In addition, glaucoma drainage device and previously failed keratoplasty might affect graft survival rates [15, 16]. The difference of preoperative from final BCVA may be explained by the limited improvement of visual acuity due to the significant comorbidities and difficult starting conditions in complex cases (e.g., multiple ocular pathologies, trauma, and/or previous surgeries). However, even in severely comorbid eyes, visual improvements were observed, and these eyes had a benefit from DMEK. The higher visual increment in eyes of group 1 can be explained by the absence of additional ocular pathologies despite a higher preoperative or baseline level of BCVA as compared to comorbid eyes (group 2).

Due to its retrospective nature, the subjective clinical judgment, and the setting at a tertiary referral center (referral of complex and difficult cases), the data available for analysis has the potential to be affected. Nevertheless, the large sample size (*n* = 361) and the separation of the data into two distinct groups aid in the assessment of the findings. Two of the comorbidities—namely, retinal detachments and severe ocular trauma—reflect a subspecialty of the tertiary referral center for retinal, sub-macular, and trauma surgery. This fact might explain the relatively high number of these two comorbidities in the eyes of group 2 (8.3%). In addition, every third eye was classified as comorbid. A center selection bias may be present in this study and could be one of its limiting factors. Moreover, the visual improvement may be due to the concomitant treatment of comorbidities (comorbidity treatment bias). Furthermore, the preferred treatment by means of DMEK in cases of intra-stromal edema and/or little to no stromal scarring represents a limiting factor (surgical treatment bias). Another limitation is the level of adherence of patients to follow-ups. Furthermore, the reduced ability to measure ECD in cases of ocular surface disease and failing grafts could also be a factor of limitation.

Based on our findings, follow-up studies are required to further evaluate the clinical outcomes in a DMEK group of patients with ocular comorbidity.

In conclusion, DMEK is a valid and proven procedure to treat endothelial disease in eyes with or without comorbidities. In cases of comorbid eyes, patient expectations should be addressed and surgical benefits explained.

## Data Availability

The datasets generated during and/or analyzed during the current study are available from the corresponding author on reasonable request.

## References

[1] Baydoun L, Müller T, Lavy I, Parker J, Rodriguez-Calvo-de-Mora M, Liarakos VS, Dapena I, Melles GRJ (2017). Ten-year clinical outcome of the first patient undergoing Descemet membrane endothelial keratoplasty. Cornea.

[2] Groeneveld-van Beek EA, Lie JT, van der Wees J, Bruinsma M, Melles GR (2013). Standardized ‘no-touch’ donor tissue preparation for DALK and DMEK: harvesting undamaged anterior and posterior transplants from the same donor cornea. Acta Ophthalmol.

[3] Dapena I, Ham L, Melles GR (2009). Endothelial keratoplasty: DSEK/DSAEK or DMEK--the thinner the better?. Curr Opin Ophthalmol.

[4] Flockerzi E, Maier P, Böhringer D, Reinshagen H, Kruse F, Cursiefen C, Reinhard T, Geerling G, Torun N, Seitz B (2018). Trends in corneal transplantation from 2001 to 2016 in Germany: a report of the DOG-section cornea and its keratoplasty registry. Am J Ophthalmol.

[5] Rodríguez-Calvo-de-Mora M, Quilendrino R, Ham L, Liarakos VS, van Dijk K, Baydoun L, Dapena I, Oellerich S, Melles GRJ (2015). Clinical outcome of 500 consecutive cases undergoing Descemet’s membrane endothelial keratoplasty. Ophthalmology.

[6] Ham L, Dapena I, Liarakos VS, Baydoun L, van Dijk K, Ilyas A, Oellerich S, Melles GRJ (2016). Midterm results of Descemet membrane endothelial keratoplasty: 4 to 7 years clinical outcome. Am J Ophthalmol.

[7] Monnereau C, Quilendrino R, Dapena I, Liarakos VS, Alfonso JF, Arnalich-Montiel F, Böhnke M, Pereira NC, Dirisamer M, Parker J, Droutsas K, Geerling G, Gerten G, Hashemi H, Kobayashi A, Naveiras M, Oganesyan O, Domingo EO, Priglinger S, Stodulka P, Silva JT, Venzano D, Vetter JM, Yiu E, Melles GRJ (2014). Multicenter study of Descemet membrane endothelial keratoplasty: first case series of 18 surgeons. JAMA Ophthalmol.

[8] Weller JM, Tourtas T, Kruse FE (2015). Feasibility and outcome of Descemet membrane endothelial keratoplasty in complex anterior segment and vitreous disease. Cornea.

[9] Yoeruek E, Bayyoud T, Hofmann J, Bartz-Schmidt KU (2013). Novel maneuver facilitating Descemet membrane unfolding in the anterior chamber. Cornea.

[10] Yoeruek E, Rubino G, Bayyoud T, Bartz-Schmidt KU (2015). Descemet membrane endothelial keratoplasty in vitrectomized eyes: clinical results. Cornea.

[11] Spaniol K, Holtmann C, Schwinde JH, Deffaa S, Guthoff R, Geerling G (2016). Descemet-membrane endothelial keratoplasty in patients with retinal comorbidity-a prospective cohort study. Int J Ophthalmol.

[12] Melles GR, Ong TS, Ververs B, van der Wees J (2006). Descemet membrane endothelial keratoplasty (DMEK). Cornea.

[13] Tappin M (2007). A method for true endothelial cell (Tencell) transplantation using a custom-made cannula for the treatment of endothelial cell failure. Eye (Lond).

[14] Melles GR, Ong TS, Ververs B, van der Wees J (2008). Preliminary clinical results of Descemet membrane endothelial keratoplasty. Am J Ophthalmol.

[15] Birbal RS, Tong CM, Dapena I, Parker JS, Parker JS, Oellerich S, Melles GRJ (2019). Clinical outcomes of Descemet membrane endothelial keratoplasty in eyes with a glaucoma drainage device. Am J Ophthalmol.

[16] Pasari A, Price MO, Feng MT, Price FW (2019). Descemet membrane endothelial keratoplasty for failed penetrating keratoplasty: visual outcomes and graft survival. Cornea.

[17] Abdelmassih Y, Dubrulle P, Sitbon C, El-Khoury S, Guindolet D, Doan S, Labetoulle M, Cochereau I, Gabison EE (2019). Therapeutic challenges and prognosis of Descemet’s membrane endothelial keratoplasty in herpes simplex eye disease. Cornea.

[18] Schlögl A, Tourtas T, Kruse FE, Weller JM (2016). Long-term clinical outcome after Descemet membrane endothelial keratoplasty. Am J Ophthalmol.

[19] Ham L, Dapena I, van Luijk C, van der Wees J, Melles GR (2009). Descemet membrane endothelial keratoplasty (DMEK) for Fuchs endothelial dystrophy: review of the first 50 consecutive cases. Eye (Lond).

[20] van Dijk K, Ham L, Tse WH, Liarakos VS, Quilendrino R, Yeh RY, Melles GRJ (2013). Near complete visual recovery and refractive stability in modern corneal transplantation: Descemet membrane endothelial keratoplasty (DMEK). Cont Lens Anterior Eye.

[21] van Dijk K, Droutsas K, Hou J, Sangsari S, Liarakos VS, Melles GR (2014). Optical quality of the cornea after Descemet membrane endothelial keratoplasty. Am J Ophthalmol.

[22] Patel HY, Ormonde S, Brookes NH, Moffatt SL, Sherwin T, Pendergrast DG, McGhee CNJ (2011). The New Zealand National Eye Bank: survival and visual outcome 1 year after penetrating keratoplasty. Cornea.

[23] Ono T, Ishiyama S, Hayashidera T, Mori Y, Nejima R, Miyata K (2017). Twelve-year follow-up of penetrating keratoplasty. Jpn J Ophthalmol.

[24] Croghan C, Chou CY, Gajree S, Ramaesh K, Anijeet D (2018). Emergency therapeutic penetrating keratoplasty in a tertiary ophthalmic care facility. Eye (Lond).

